# The effect of cisatracurium infusion on the energy expenditure of critically ill patients: an observational cohort study

**DOI:** 10.1186/s13054-020-2744-7

**Published:** 2020-02-03

**Authors:** W. A. C. Koekkoek, Y. A. Menger, F. J. L. van Zanten, D. van Dijk, A. R. H. van Zanten

**Affiliations:** 1 Department of Intensive Care Medicine, Gelderse Vallei Hospital, Willy Brandtlaan 10, 6716 RP Ede, The Netherlands; 2 Department of Intensive Care Medicine, Diakonessenhuis Utrecht, Bosboomstraat 1, 3582 KE Utrecht, The Netherlands; 3 Department of Intensive Care Medicine, Gelderse Vallei Hospital, University Medical Center Amsterdam, Willy Brandtlaan 10, 6716 RP Ede, The Netherlands; 4 Department of Intensive Care Medicine, University Medical Centre Utrecht, Heidelberglaan 100, 3508 GA Utrecht, The Netherlands

**Keywords:** Energy expenditure, Overfeeding, Neuromuscular blocking agent

## Abstract

**Background:**

Both overfeeding and underfeeding of intensive care unit (ICU) patients are associated with worse outcomes. A reliable estimation of the energy expenditure (EE) of ICU patients may help to avoid these phenomena. Several factors that influence EE have been studied previously. However, the effect of neuromuscular blocking agents on EE, which conceptually would lower EE, has not been extensively investigated.

**Methods:**

We studied a cohort of adult critically ill patients requiring invasive mechanical ventilation and treatment with continuous infusion of cisatracurium for at least 12 h. The study aimed to quantify the effect of cisatracurium infusion on EE (primary endpoint). EE was estimated based on ventilator-derived VCO_2_ (EE in kcal/day = VCO_2_ × 8.19). A subgroup analysis of septic and non-septic patients was performed. Furthermore, the effects of body temperature and sepsis on EE were evaluated. A secondary endpoint was hypercaloric feeding (> 110% of EE) after cisatracurium infusion.

**Results:**

In total, 122 patients were included. Mean EE before cisatracurium infusion was 1974 kcal/day and 1888 kcal/day after cisatracurium infusion. Multivariable analysis showed a significantly lower EE after cisatracurium infusion (MD − 132.0 kcal (95% CI − 212.0 to − 52.0; *p* = 0.001) in all patients. This difference was statistically significant in both sepsis and non-sepsis patients (*p* = 0.036 and *p* = 0.011). Non-sepsis patients had lower EE than sepsis patients (MD − 120.6 kcal; 95% CI − 200.5 to − 40.8, *p* = 0.003). Body temperature and EE were positively correlated (Spearman’s rho = 0.486, *p* < 0.001). Hypercaloric feeding was observed in 7 patients.

**Conclusions:**

Our data suggest that continuous infusion of cisatracurium in mechanically ventilated ICU patients is associated with a significant reduction in EE, although the magnitude of the effect is small. Sepsis and higher body temperature are associated with increased EE. Cisatracurium infusion is associated with overfeeding in only a minority of patients and therefore, in most patients, no reductions in caloric prescription are necessary.

## Background

Targeting optimal nutrition concerning energy goals is essential in critically ill patients, as both underfeeding and overfeeding have been associated with increased morbidity and mortality [1]. Ideally, the target is based on energy expenditure (EE). However, due to the pathophysiological response to critical illness, iatrogenic interventions, and differences in body composition, EE is highly variable in and between critically ill patients [2]. Frequent monitoring of EE may circumvent this problem and help to adjust the optimal amount of calories on an individual basis. At present, indirect calorimetry is considered the gold standard. However, frequently, this technique is not available and often unfeasible [3].

To optimize nutritional targets without frequent monitoring of EE, it is essential to know which factors are associated with either an increase or decrease in EE.

Specific conditions expected to influence EE have been studied such as sepsis [4–6], burns [4, 7], trauma [4, 8], cerebrovascular accidents [4, 9], pregnancy [10], body temperature [4], administration of sedatives [11], and therapeutic hypothermia [4, 12]. An increased EE has been reported in patients with sepsis, trauma, burns, fever, and pregnancy. Therapeutic hypothermia and the administration of sedatives are associated with a decrease in EE [4]. However, limited information is available on the effects of neuromuscular blocking agents (NMBAs) on EE. Furthermore, it is not known whether NMBA administration affects the EE in sepsis patients similarly compared with non-sepsis patients and in relation to the baseline temperature.

This study aimed to quantify the effect of cisatracurium infusion on EE of adult critically ill patients. Also, we analyzed the effects of body temperature and sepsis on EE. Secondary endpoint was hypercaloric feeding as a consequence of muscle relaxation.

## Materials and methods

We performed a retrospective observational study in patients treated with cisatracurium at the mixed medical-surgical adult intensive care unit of the Gelderse Vallei Hospital, Ede, The Netherlands, between January 1, 2011, and October 31, 2016. Patients were included when they met with the following inclusion criteria: adult critically ill patients (≥ 18 years) requiring invasive mechanical ventilation and treatment with cisatracurium for at least 12 h.

Exclusion criteria were pregnancy, hypothermia induced by therapeutic temperature management, burns, and malignant hyperthermia because these conditions have a substantial effect on EE. Patients were also excluded when data on VCO_2_ were incomplete. In patients with multiple ICU admissions during the study period, data from readmissions were excluded. An ICU admission was considered readmission when the patient was admitted within 6 months from the primary ICU admission.

### Administration of cisatracurium

Cisatracurium is the NMBA of choice for sustained neuromuscular blockade during critical illness in Gelderse Vallei Hospital. Cisatracurium was administered when indicated according to the international clinical practice guidelines for the sustained neuromuscular blockade in the adult critically ill patient [13]. An infusion was started at doses of 3 μg/kg per minute and then adjusted by assessment of the train-of-four (TOF) using a peripheral nerve stimulator (TOF-watch® S, Dublin, Ireland). According to the hospital protocol, TOF measurements were performed every hour, and dosage adjustments were made to achieve a TOF level of 1 or lower. The electrodes of the TOF-watch® were placed on the other wrist daily to prevent skin lesions.

### Outcome measures

The primary endpoint was the total EE, expressed as kcal/day, which was measured before and during cisatracurium infusion. Indirect calorimetry was not routinely available during the study period. EE was, therefore, estimated by an adjusted version of Weir’s equation using the ventilator-derived VCO_2_ (EEVCO_2_). EEVCO_2_ = 3.941 × VCO_2_(L/min) / respiratory quotient + 1.11 × VCO_2_(L/min) × 1440. The respiratory quotient was considered to be a fixed value of 0.86 [14–16]. The mechanical ventilator measured the VCO2 (Hamilton-S1, Hamilton Medical AG, Bonaduz, Switzerland), and every minute, data are automatically sent to our electronic patient data management system (MetaVision; iMDsoft MetaVision®, Tel Aviv, Israel). For each patient, the VCO_2_ was collected during the 12 h before and during the 12 h after the start of cisatracurium infusion. When patients were not admitted to the ICU 12 h before the start of cisatracurium infusion, the parameters of the available hours were used. The EEVCO_2_ was calculated every 2 h using the mean VCO_2_ measurements from the previous 2 h.

Secondary endpoint was hypercaloric feeding (> 110% of EE) after cisatracurium infusion. We also evaluated ICU length of stay (LOS) and in-hospital mortality in patients receiving hypercaloric versus regular or hypocaloric feeding.

### Calculation of nutritional goals

The World Health Organization/Food and Agricultural Organization of the United Nations (WHO/FAO) formulas were used to calculate caloric and protein targets by our computerized feeding protocol [17]. According to BMI, the actual (BMI < 27), corrected (BMI 27–30; regression to BMI of 27), or ideal body weight (BMI > 30; regression to BMI 21 in women and BMI 22.5 in men) was used. An addition to the resting EE (REE) of 20% was used to correct for disease activity [18].

### Data collection

Most parameters were routinely collected into an extensive ICU database during standard clinical care. Data extraction was performed using SAS Enterprise Guide queries (version 7.12HF1) searching our Patient Data Management System (MetaVision; iMDsoft MetaVision®, Tel Aviv, Israel, and neoZIS®, Electronic Medical Record, MI Consultancy, Katwijk, The Netherlands). Data to calculate the Charlson Comorbidity Index (CCI) [19] were obtained from the quality management system for hospital mortality registration. Data verification was performed manually. Collected data were de-identified and stored on a secure hospital computer. There were no identifiable paper documents.

### Data analysis and statistical considerations

Descriptive data are reported as means and standard deviation (SD) or median and interquartile range in case of skewed distributions, or as frequencies and percentages when appropriate. For the primary analysis, comparing the EE before and after cisatracurium infusion, a general linear mixed model analysis for repeated measures was performed with an autoregressive covariance structure. In this analysis, we corrected for body temperature, sedative and noradrenaline dosages, pH, PEEP, and FiO_2_ and repeated measurements.

We performed a subgroup analysis of septic and non-septic patients. We also evaluated the effects of body temperature on EE with the Pearson or Spearman rank correlation tests. The effects of sepsis on EE were analyzed through general linear mixed models, correcting for the following confounders: cisatracurium, temperature, NUTRIC score, gender, BMI, admission type, and repeated effects. Finally, we evaluated the effect of hypercaloric feeding vs. normocaloric and hypocaloric feeding on in-hospital mortality and ICU length of stay (LOS) by chi-square test and one-way ANOVA, respectively. A *p* value of < 0.05 was considered statistically significant.

The data analyses were performed using IBM corp. SPSS statistics for Windows (version 24.0, released 2015 New York, USA).

## Results

### Patients

**Fig. 1:**
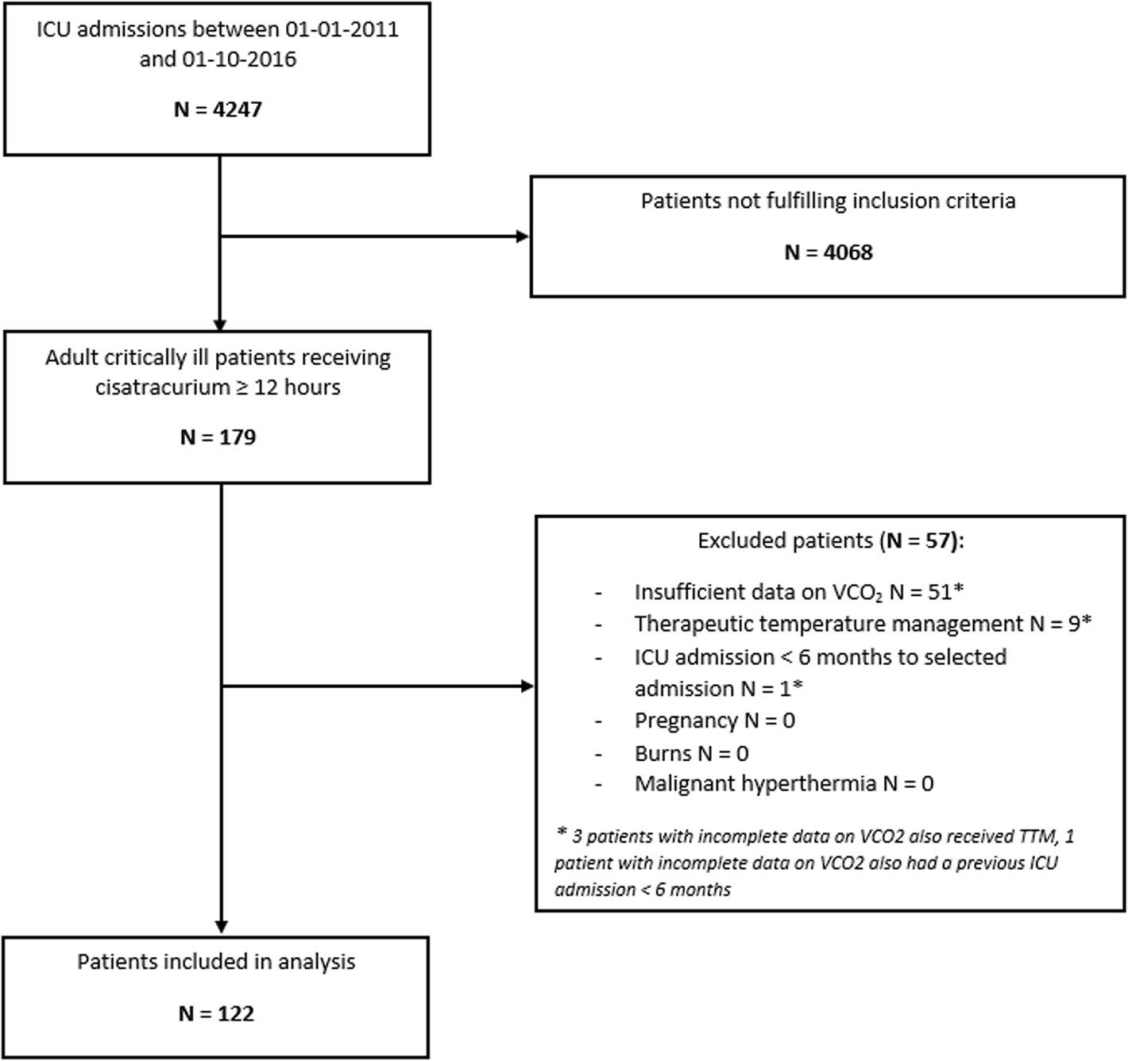
Flowchart

During the study period, 4247 patients were admitted to the ICU, of which 179 received cisatracurium for at least 12 h and therefore were eligible for inclusion. We excluded 57 patients according to the exclusion criteria. In total, 122 patients were enrolled in this study (Fig. 1).

Baseline characteristics and nutritional parameters are shown in Tables 1, 2, and 3. The median age was 65.5 years, and 36.1% were female. The median SOFA and APACHE II scores on admission were 8 and 22, respectively. Most patients were septic (58.2%) and admitted to the ICU because of medical reasons (73%). A median ICU and hospital LOS of 15 and 23 days were found. The in-hospital mortality was 28.7%.

**Table 1:** Baseline characteristics

		Total (*n* = 122)
Gender (female)	*N* (%)	44 (36.1)
Age (years)	Median [IQR]	66 [55–73]
BMI on admission (kg/m^2^)	Median [IQR]	27.6 [24.0–31.0]
▪ Malnourished (< 18.5)	*N* (%)	3 (2.5)
▪ Normal (18.5–24.9)		33 (27.0)
▪ Overweight (25–29.9)		44 (36.1)
▪ Obese (30–34.9)		26 (21.3)
▪ Morbidly obese (> 35)		16 (13.1)
Admission type	*N* (%)	
▪ Medical		89 (73.0)
▪ Emergency surgery		16 (13.1)
▪ Elective surgery		17 (13.9)
Sepsis	*N* (%)	72 (59.0)
Charlson Comorbidity Index	Median [IQR]	4 [2–5]
SOFA score on admission	Median [IQR]	8 [5–9.5]
APACHE II score on admission	Median [IQR]	21.5 [19 – 26.25]
ICU length of stay (days)	Median [IQR]	15 [8–26.5]
Hospital length of stay (days)	Median [IQR]	23 [12–42]
In-hospital mortality	*N* (%)	36 (29.5)

*BMI* body mass index, *IQR* interquartile range, *SOFA* Sequential Organ Failure Assessment, *APACHE* acute physiology and chronic health evaluation, *ICU* intensive care unit

**Table 2:** Baseline characteristics before and after cisatracurium administration

		Before cisatracurium (*n* = 122)	After cisatracurium (*n* = 122)	*p* value
PEEP (cmH_2_O)	Mean ± SD	9.8 ± 3.0	10.6 ± 3.9	0.012
FiO_2_ (%)	Median [IQR]	49 [40–59]	44.5 [36–55]	0.008
Noradrenalin (μg/kg/min)	Mean ± SD	0.16 ± 0.64	0.33 ± 1.25	0.178
Propofol (mg/h)	Mean ± SD	44.9 ± 73.1	42.2 ± 70.8	0.570
Midazolam (mg/h)	Mean ± SD	7.5 ± 5.3	9.0 ± 5.8	< 0.001
Morphine (mg/h)	Mean ± SD	1.2 ± 1.3	1.5 ± 1.4	< 0.001
pH	Mean ± SD	7.31 ± 0.11	7.32 ± 0.10	0.600
TOF	Median [IQR]	NA	0 [0–1]	NA

*PEEP* positive end-expiratory pressure, *FiO*_*2*_ fraction of inspired oxygen, *TOF* train of four, *NA* not applicable

**Table 3:** Nutritional parameters

		Total (*n* = 122)
NUTRIC score at admission	Median [IQR]	6 [4–7]
▪ Low risk (0–4 points)	*N* (%)	35 (29.9)
▪ High risk (5–9 points)	*N* (%)	82 (71.1)
Nutritional route^a^	*N* (%)	
▪ Enteral		91 (91.9)
▪ Parenteral		6 (6.1)
▪ Both		2 (2.0)
Average caloric intake (kcal/day)^b^	Mean (±SD)	831 (612)

*NUTRIC* Nutrition Risk in Critically ill [20], *IQR* interquartile range, *SD* standard deviation

^a^Nutritional route during the first 24 h of ICU admission

^b^Average caloric and protein intake during the first day of cisatracurium administration (kcal/day)

### Primary outcome

**Fig. 2:**
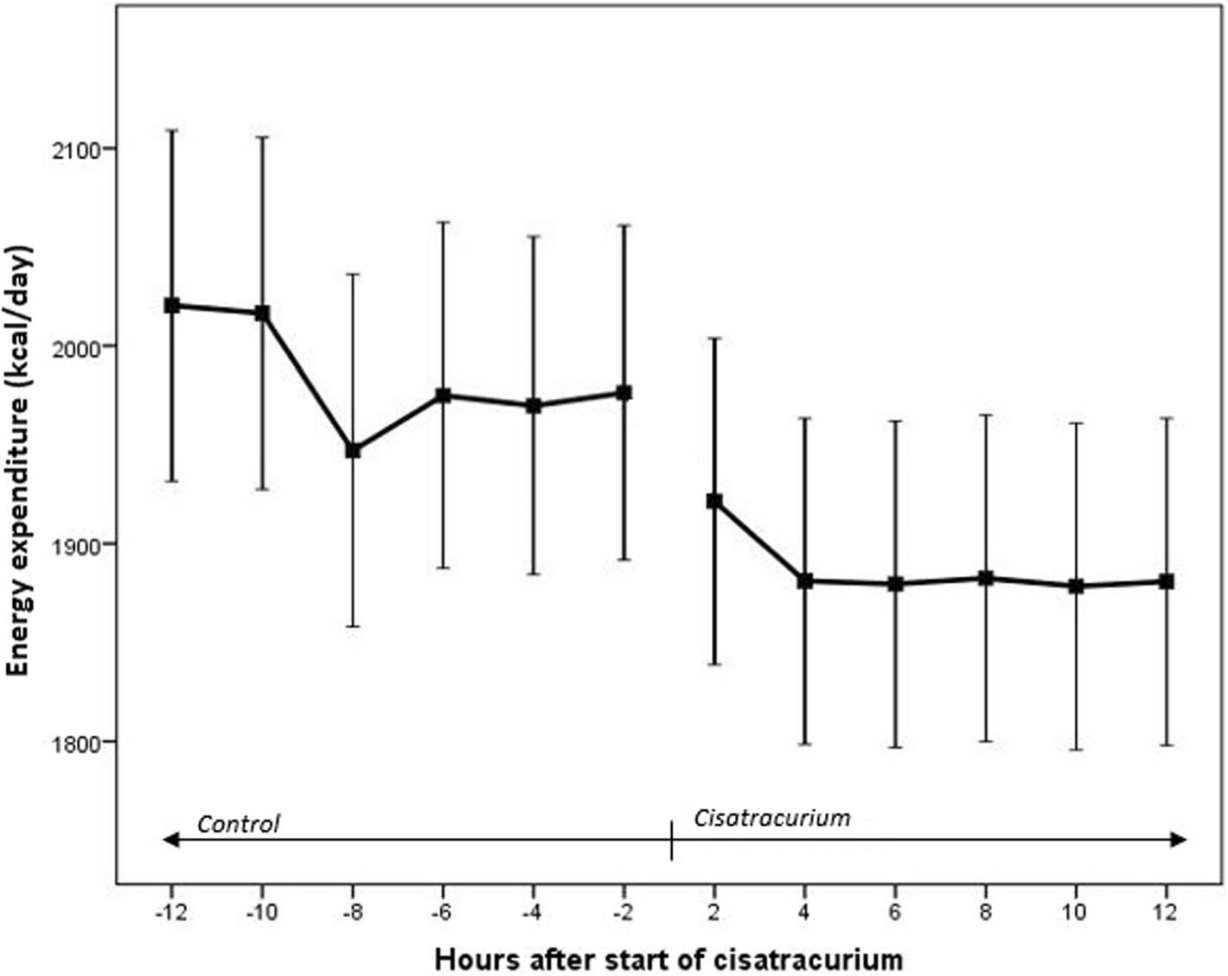
Energy expenditure before and during continuous cisatracurium infusion

The mean EE was 1974 kcal/day before cisatracurium infusion (= control period) and 1888 kcal/day during cisatracurium infusion resulting in a mean difference of − 85.9 kcal (95% CI − 151.8 to − 20.0; *p* = 0.011). After correction for body temperature, sedative and noradrenalin dosages, pH, PEEP, and FiO_2_ in mixed model multivariable analysis, the significant treatment effect of cisatracurium on EE persisted, with a mean difference of − 132.0 kcal (95% CI − 212.0 to − 52.0; *p* = 0.001). Cisatracurium significantly lowered EE by 6.6% (95% CI 2.6–10.6%). The results are depicted in Fig. 2.

#### Subgroup analysis of sepsis patients

In the subgroup of sepsis patients, cisatracurium reduced EE from 2058 kcal/day to 1932 kcal/day (mean difference of − 125.7 kcal; 95% CI − 243.0 to − 8.4; *p* = 0.036). In the subgroup of non-sepsis patients, cisatracurium reduced EE from 1932 kcal/day to 1795 kcal/day (mean difference − 137.2 kcal; 95% CI − 243.0 to − 31.4; *p* = 0.011). In both analyses, adjustment for body temperature, sedative and noradrenaline dosages, pH, PEEP, and FiO_2_ were performed.

### Effect of body temperature on EE

**Fig. 3:**
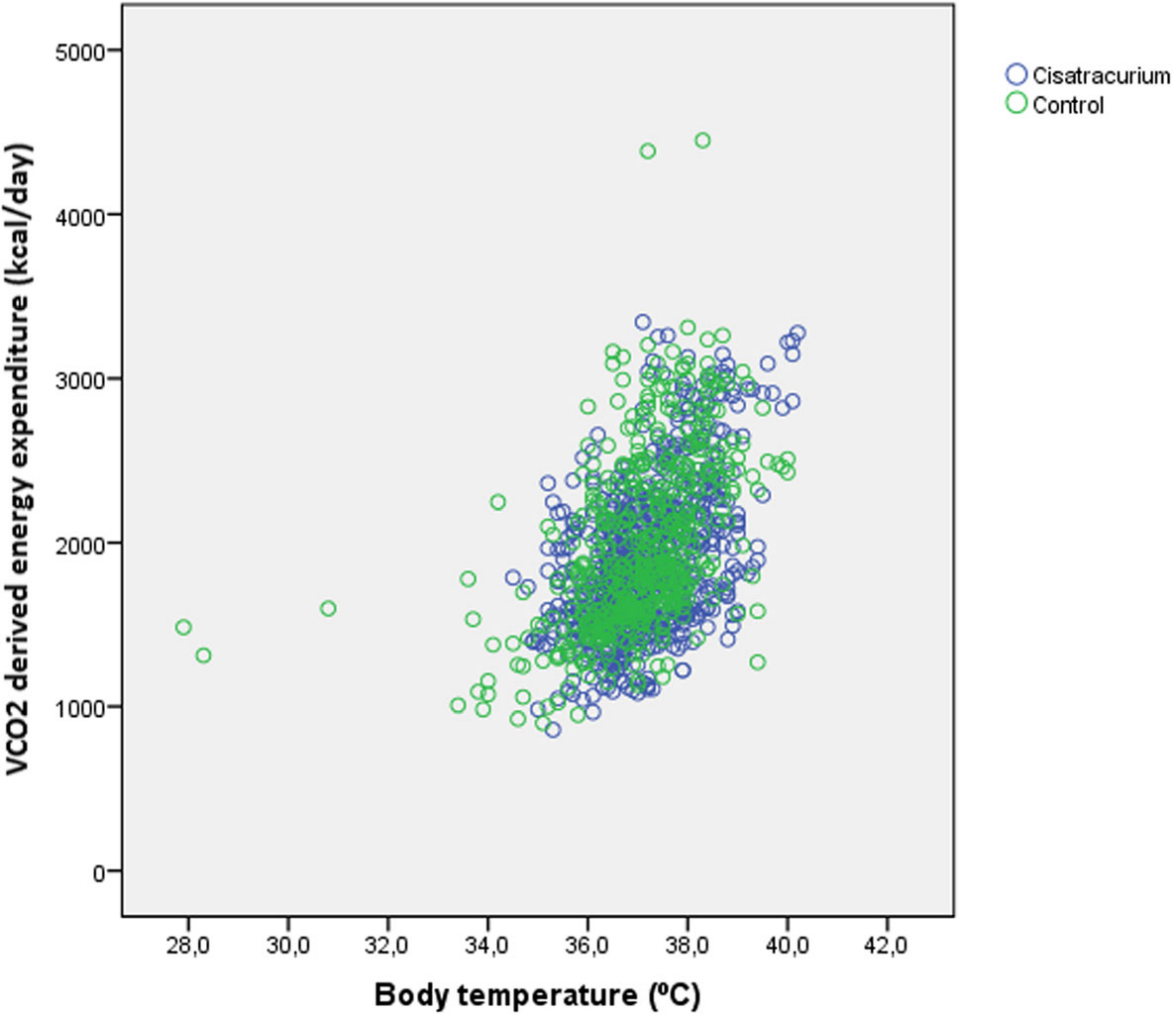
Association between body temperature and energy expenditure

A significant non-linear positive association between body temperature and EE was found (Spearman’s rho = 0.486, *p* < 0.001; Fig. 3).

### Effect of sepsis on EE

Mean EE was 1805 kcal (95% CI 1721–1888) in non-septic patients and 1909 kcal (95% CI 1838–1978) in septic patients (*p* = 0.062). In mixed-model multivariable analysis, a significantly lower EE was observed in non-septic patients than in septic patients (mean difference − 120.6 kcal, 95% CI − 200.5 to − 40.8; *p* = 0.003).

### Hypercaloric feeding

Only seven patients (5.7%) received > 110% of their caloric target (estimated by EEVCO_2_) on the first day of cisatracurium infusion. Twenty patients (16.4%) received between 80 and 110% of their caloric target, while 95 patients (77.9%) were fed hypocalorically (< 80% of caloric target). Because of the small number of patients with hypercaloric intake, no associations between hypercaloric intake and ICU LOS or mortality were calculated.

## Discussion

We studied the effect of cisatracurium infusion on EE in a cohort of 122 adult critically ill patients. Cisatracurium infusion lowered EE as estimated by the VCO_2_ method by 6.6%.

NMBAs act by interfering with the binding of acetylcholine to the motor endplate in the synaptic cleft of the neuromuscular junction, thereby ultimately preventing muscle contraction. Indications for the continuous infusion of NMBAs during critical illness comprise severe acute respiratory distress syndrome (ARDS) (PaO_2_/FiO_2_ < 150), overt shivering during therapeutic hypothermia, and other life-threatening situations associated with profound hypoxemia, respiratory acidosis, or hemodynamic compromise in case of failure of other measures such as deep sedation [13]. Cisatracurium is one of the most widely used NMBAs for continuous infusion as it can also be used in patients with hepatic or renal insufficiency [21].

Due to the blocking of muscle contractions and as a consequence of the subsequent lower muscular heat production, NMBAs should conceptually reduce EE. However, this hypothesis has not been studied in ICU patients with the previously described indications for the use of continuous NMBA infusion. Overall, only one earlier study has evaluated the effects of NMBAs on EE in adults, reporting a significant increase in EE of 18.6% after discontinuation of pancuronium in patients with severe head injury [22]. Additionally, one study investigated the effects of NMBA infusion (vecuronium, pancuronium, and atracurium) in 20 critically ill children reporting a significant reduction of 10.3% of EE 1 h after infusion of NMBAs [23].

### Effect of body temperature on EE

We observed a non-linear positive association between body temperature and EE. Four small previous studies reported an association between body temperature and EE in critically ill patients [4, 24, 25]. A reduction of 6.6% of EE per 1 °C decrease at temperatures below 36 °C and an increase of 8.2% per 1 °C at temperatures above 37 °C have been reported [24, 25].

### Effect of sepsis on EE

We observed a higher EE in septic patients than in non-septic patients. This was in line with our expectations based on previous studies in which EE in septic patients was 102–198% of EE in non-septic patients [4]. However, a recent observational study in 205 patients found no differences in EE between septic and non-septic patients (1434 vs. 1430 kcal/day) [20].

### Strengths and weaknesses

This is the largest cohort of critically ill patients in which the effects of NMBAs on EE have been studied. The effects of NMBAs, especially cisatracurium, in this specific patient population have not been studied before. A large number of patient variables were available with few missing data, providing enough data to perform rigorous multivariable and repeated measure analyses.

However, our study has several limitations. Indirect calorimetry was not routinely available during the study period. Therefore, EE was calculated using VCO_2_ obtained from the mechanical ventilator. Calculation of EE from VCO_2_ has been demonstrated to be more accurate than predictive equations, but less than indirect calorimetry. Finally, limitations related to the retrospective design may potentially have introduced bias and residual confounding.

### Clinical implications

As cisatracurium reduces EE, reduction of caloric intake after the start of NMBAs should be considered, especially in those patients that are on full feeding or considered to reach this target soon, because they are at risk of hypercaloric feeding and associated harm. Before we designed the study, we expected, due to the drop in EE induced by the NMBA, that some of the patients would be overfed. Based on the results, we noticed that a reduction of EE by NMBA could induce an almost 10% overfeeding risk in individual patients. In daily practice, this did not occur as the patients were not on nutrition target. Thus, for most patients, adjustment may not be necessary as in our analysis the reduction of EE found was only 6.6% and hypercaloric feeding was only present in 5.7%, while most other patients were fed (77.9%) hypocalorically after initiation of cisatracurium infusion.

Although not the focus of our present study, it should be noted that the recent ROSE trial, studying the effect of early neuromuscular blockade (48-h continuous infusion of cisatracurium) with concomitant heavy sedation, compared with usual care, did not result in a significant mortality difference at 90 days in patients with moderate to severe acute respiratory distress syndrome in contrast to an earlier RCT [26, 27]. This trial was stopped early at the second interim analysis for futility. This study may lead to reevaluation of the use of NMBAs in severe respiratory failure.

## Conclusions

Our data suggest that continuous infusion of cisatracurium in mechanically ventilated ICU patients is associated with a significant reduction in EE as estimated by the VCO_2_ method, although the magnitude of the effect is small. Sepsis and higher body temperature are associated with increased EE. Cisatracurium infusion is associated with overfeeding in only a minority of patients, and therefore, in most patients no reductions in caloric prescription are necessary.

## Data Availability

The datasets used and/or analyzed during the current study are available from the corresponding author on reasonable request.

## Abbreviations

APACHE: Acute physiology and chronic health evaluation
ARDS: Acute respiratory distress syndrome
BMI: Body mass index
CCI: Charlson Comborbidity Index
EE: Energy expenditure
EEVCO_2_: Energy expenditure calculated from ventilator-derived VCO_2_
FAO: Food and Agriculture Organization
FiO_2_: Fraction of inspired oxygen
ICU: Intensive care unit
LOS: Length of stay
NMBA: Neuromuscular blocking agent
NUTRIC: Nutrition Risk in Critically ill
PEEP: Positive end-expiratory pressure
SD: Standard deviation
SOFA: Sequential Organ Failure Assessment
TOF: Train of four
WHO: World Health Organization
